# Developing a Digital Therapeutic for Alcohol Reduction – a Pilot Study of Curb, an App for People Who Want to Change Their Relationship With alcohol

**DOI:** 10.1192/bjo.2024.205

**Published:** 2024-08-01

**Authors:** Helen Anderson, Romayne Gadelrab, David McLaughlan, Faith Matcham

**Affiliations:** 1 South London and the Maudsley NHS foundation Trust, London, United Kingdom; 2 Curb Health, London, United Kingdom; 3 Curb Health, London, United Kingdom; 4 The Priory Group, London, United Kingdom; 5 University of Sussex, Brighton, United Kingdom

## Abstract

**Aims:**

1. Evaluate user engagement.
2. Evaluate safety.
3. Evaluate efficacy.
  - To reduce drinking.
  - To address comorbid symptoms of anxiety and depression.

**Methods:**

*Study Population*

Participants applied via social media, identifying as individuals seeking to change relationships with alcohol. Inclusion criteria: Aged >18, Alcohol Use Disorder Identification Test (AUDIT) score of 8–16, no history of withdrawal symptoms, or AUDIT score 16–20 but already abstinent for >14 days. Participants who had already been abstinent for >30 days excluded.

*The Programme*

Participants given unlimited access to Alma mobile application (app) for 4 weeks. Programme consisted of daily pledge to cut down drinking, drink diary to record alcohol use, weekly feedback on Generalised Anxiety Disorder-7 (GAD-7) scores and Patient Health Questionnaire-9 (PHQ-9) depression scores, unlimited access to mindfulness videos to manage cravings.

*Statistical analysis*

Mixed-effects linear regression used for analysis.

**Results:**

57 people volunteered for pilot study. 31 eligible to participate.

*Engagement*

Progressive weeks of programme showed attrition in user numbers. By end of 4-week programme, 77% (24/31) remained, 58% (18/31) submitted all data.

*Safety*

All participants asked if they had experienced no harm or distress from using app. 25 participants answered, 100% (25/25) responded “no”.

*Efficacy*

Self-reported capability to reduce drinking significantly increased over time (mean increase from baseline +0.3; p = 0.007). At week 4, 8/17 (47.1%) said that Alma had helped them cut down drinking a lot, and a further 8/17 (47.1%) said it helped them cut down a bit.

There was a trend for units drunk on the heaviest drinking day to reduce over time (−0.48 units) and total weekly consumption of units to reduce (−1.01 units), however not statistically significant. There was no trend for drinking days per week to reduce over time.

There was a significant reduction in PHQ-9 scores over time (−1.03; p < 0.001) and significant reduction in GAD-7 scores (−0.69; p < 0.001).

A total of 22/24 (92%) respondents said they would recommend Alma to friends and family, 1/24 (4%) would not.

**Conclusion:**

- Relatively high engagement with Alma compared with similar digital products.
- Pilot study suggests Alma is acceptable, safe and shows potential efficacy in helping reduce alcohol intake and comorbid anxiety/depression, however interpretation limited by small sample size.
- Next steps will be to widen user-base to facilitate larger studies, and gain further insights into factors influencing relapses by studying associations with health-related data from wearable devices and other user inputs.

